# A Novel Scoring System for Predicting Mortality, Morbidity, and Functional Outcomes in Patients Following Below-Knee Amputation: A Retrospective Study

**DOI:** 10.7759/cureus.80967

**Published:** 2025-03-21

**Authors:** Goker Yurdakul, Fatih Golgelioglu, Haci Ali Olcar, Davut Aydin, Satuk Bugrahan Yinanc, Murat Korkmaz

**Affiliations:** 1 Department of Orthopedics and Traumatology, Yozgat Bozok University Faculty of Medicine, Yozgat, TUR; 2 Department of Orthopedics and Traumatology, Haymana State Hospital, Ankara, TUR; 3 Sarıkaya School of Physiotherapy and Rehabilitation, Yozgat Bozok University, Yozgat, TUR

**Keywords:** follow-up, mobilization, morbidity, scoring method, survival analysis

## Abstract

Objective

Patients undergoing below-knee amputation may experience considerable postoperative mortality risk, particularly in the presence of comorbid conditions. The aim of this study was to present a newly developed risk index and scoring system to predict one-year mortality, morbidity, and functional independence in patients undergoing below-knee amputation.

Materials and methods

One-year postoperative follow-up data were obtained retrospectively from 30 patients who underwent below-knee amputation at our clinic. A novel scoring system was developed using variables including age, preoperative systemic diseases, diabetic foot infection, previous extremity surgery, postoperative mobilization time, and early complications. Survival analysis was performed, and functional independence was assessed using the Katz Activities of Daily Living Scale (Katz scores). The relationship between patients' survival status and Katz scores with the developed risk index was statistically evaluated.

Results

The average age of the patients was 71.7 years. Survival analysis indicated that higher scores on the newly developed index were significantly associated with increased mortality and morbidity (p<0.05). There was also a strong negative correlation between patients' scores and Katz scores (r=-0.757; p<0.001), indicating that patients with higher risk scores experienced poorer functional outcomes.

Conclusions

This retrospective study introduced a novel scoring system that reliably predicts functional independence in patients following below-knee amputation. However, its accuracy in predicting mortality and morbidity remains limited. Further refinement and validation in larger patient populations are required to enhance predictive accuracy and clinical applicability.

## Introduction

In 2005, approximately 1.6 million people in the United States were living with limb loss, and this number is projected to increase to 3.6 million by 2050 [1]. Below-knee amputation is a high-risk surgical procedure associated with a significant risk of mortality [2,3]. Despite numerous studies investigating the risk factors for mortality and morbidity following below-knee amputation, the complexity and interactions between these factors present challenges in postoperative management and make it difficult to accurately assess individual mortality risk [4].

A risk index to predict postoperative mortality could significantly improve patient care and potentially improve survival. Currently, there is no consensus or standardized risk index or scoring system in the literature for assessing mortality and morbidity after below-knee amputation [3].

A numerical risk index would be clinically useful, providing a method of scoring patients based on their risk factors and using the scoring method to predict individual postoperative mortality rates. The aim of the study was to present a newly developed risk index that predicts one-year mortality, morbidity, and functional daily ability in patients undergoing below-knee amputation.

## Materials and methods

The current research utilized a retrospective study, with patient medical records obtained through appropriate approval from the Yozgat Bozok University Ethics Committee (approval number: 2017-KAEK-189-2021.11.17_02 189; date: 17/11/2021). The study completely adhered to the ethical guidelines outlined in the Helsinki Principles. We evaluated 30 patients who underwent below-knee amputation between December 2017 and December 2021 during one year.

The study applied specific exclusion and inclusion criteria to select the appropriate patient population. Exclusion criteria included patients with less than one year of clinical follow-up, those with incomplete medical records, or those who underwent neoplastic, bilateral, or traumatic amputations. Since the study specifically focuses on below-knee amputations due to non-traumatic causes, patients with amputations at other levels or resulting from trauma were not included. This decision was made to ensure a more homogeneous patient population and to avoid confounding factors related to trauma-associated physiological and prognostic differences. The inclusion criteria were limited to patients who underwent non-traumatic below-knee amputations, were over the age of 18 at the time of their first amputation, and had complete follow-up data.

All patients underwent a standardized below-knee amputation procedure in the supine position without the use of a tourniquet. Given the retrospective nature of the study, surgical details were not a primary focus; however, intraoperative tissue viability was noted in all cases. Postoperatively, patients received standard thromboprophylaxis, anti-inflammatory treatment, and analgesia, as these factors could potentially influence recovery and outcomes.

The development of the scoring system was guided by factors frequently reported in the literature as being associated with postoperative mortality [3,5-16]. These factors included age, preoperative comorbid systemic diseases, postoperative mobilization, metabolic disorders, and postoperative hypoxia, which is defined as an oxygen saturation level below 90% due to respiratory or circulatory insufficiency after surgery. Each of these factors was incorporated into the scoring system based on prior studies that demonstrated an increased risk of postoperative complications with advancing age (Table 1). Specifically, age was categorized as follows: 0 points for patients under 65 years, 1 point for those aged 65-79, and 2 points for patients over 79 years, in accordance with established risk stratification models [3,6,13]. 

**Table 1:** Parameters used in the development of the scoring system

Scoring method	0 point	1 point	2 points
Age	<65 years old	65-79 years old	>79 years old
Preoperative systemic disease		1 point for each systemic disease	
Mobilization	Early, within 24 hours after the operation	Late, >24 hours after the operation	Bedridden, no mobilization
Postoperative hypoxia or metabolic disorder		1 point for each	

Preoperative comorbid systemic diseases

One point was assigned for each of the following conditions: chronic obstructive pulmonary disease, hypertension, diabetes mellitus, previous myocardial infarction, previous stroke, and previous lower extremity infection [8,14].

Postoperative mobilization

Early functional mobilization, with the assistance of a walking aid, was initiated immediately after the below-knee amputation for patients who could tolerate it. Early mobilization was defined as the ability to mobilize within the first 24 hours postoperatively, late mobilization referred to mobilization occurring later, and immobilization was defined as bedridden status. The scoring was assigned as follows: early mobilization (0 points), late mobilization (1 point), and immobilization (bedridden) (2 points) [5,15].

Postoperative hypoxia or metabolic disorder

Hypoxia was defined as an oxygen saturation below 90% or a need for supplemental oxygen beyond baseline requirements within the first 72 hours postoperatively. Metabolic disorders included significant electrolyte imbalances (e.g., serum sodium <130 or >150 mmol/L, potassium <3.0 or >5.5 mmol/L) or acid-base disturbances (pH <7.30 or >7.45) requiring medical intervention. Each occurrence was assigned 1 point [11,16].

The Katz score was utilized to assess an individual's ability to perform activities of daily living [17]. This test evaluates functional status by measuring performance in six essential activities: bathing, dressing, toileting, transferring, personal care, and feeding. Each activity is scored with a yes/no response, indicating whether the individual can perform the task independently. A Katz score of 6 indicates full independence, a score of 4 suggests moderate functional ability, and a score of 2 or below indicates severe functional impairment.

Statistical analysis

Data were analyzed using IBM SPSS Statistics for Windows, V. 27.0 (IBM Corp., Armonk, NY, USA). Descriptive statistical methods were initially employed. To assess differences among the clinical characteristics, the Mann-Whitney U test was employed. Receiver operating characteristic (ROC) analysis was employed to assess the predictive accuracy of the developed scale in estimating one-year mortality. The ROC curve demonstrates the sensitivity and specificity at various cutoff points, reflecting the scale's ability to differentiate between outcomes. The area under the curve (AUC) is used to quantify this accuracy, with values ranging from 0.5 (indicating no discriminatory ability) to 1.0 (indicating perfect discrimination). Spearman's correlation analysis was performed to evaluate the relationship between the developed scale and the Katz index of independence in activities of daily living. In interpreting Spearman's correlation coefficient (r), values between 0.00 and 0.10 indicate a negligible correlation, 0.10 and 0.39 indicate a weak relationship, 0.40 and 0.69 indicate a moderate relationship, 0.70 and 0.89 indicate a strong relationship, and 0.90 and 1.0 indicate a very strong relationship [18]. Since five patients passed away within the first three months, they were excluded from the analysis of Katz scores. This adjustment was made to maintain the accuracy and relevance of the dataset when evaluating functional independence at the three-month follow-up.

## Results

**Figure 1:**
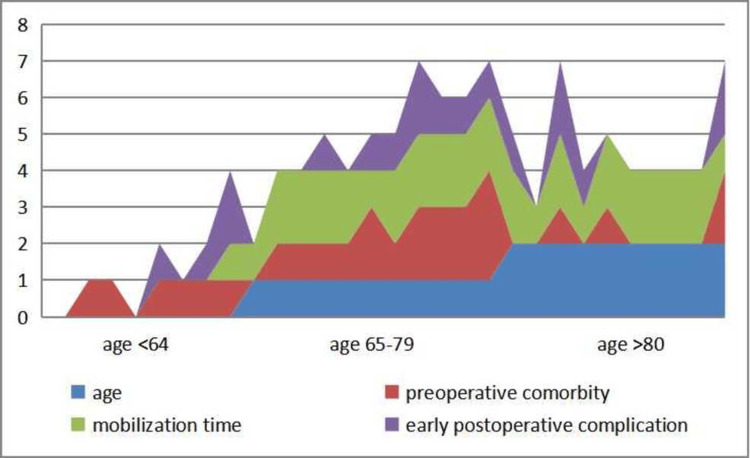
The four parameters that make up our score are age, preoperative disease, postoperative mobilization time, and complications that develop in the early postoperative period. The relationship between our score consisting of these parameters and one-year postoperative survival and the Katz score at three months postoperatively was investigated

The mean age of the 30 patients who underwent below-knee amputation was 71.7 years. Prior to surgery, 19 patients had a history of limb-related surgical debridement, foot infection, or additional systemic disease. The scoring system developed for this study incorporates key factors such as age, mobilization status, preoperative comorbidities, and early postoperative complications. The distribution of these factors in the scoring method is illustrated in Figure 1.

A total of 16 patients were successfully mobilized postoperatively, with eight mobilized early and eight mobilized late. However, 14 patients remained immobile (bedridden) after surgery (Table 2). Metabolic disorders or hypoxia, classified as early postoperative complications, was observed in 14 patients. Within one year, 15 out of the 30 patients had passed, while the remaining 15 survived beyond the one-year follow-up period. Additionally, six patients experienced a myocardial infarction, and four had a stroke during the first year postoperatively; of these 10 patients, eight passed away within the year. The surviving patients exhibited severe functional impairment, with an average Katz score of 1.69 at three months. In our scoring system, the average score was 3.73.

**Table 2:** Demographic values of the patients

Patients' demographic values	
Age	71.7±1.26
The mean of the scoring we defined	3.73
Preoperative comorbidity	19
Mobilization time	<8 (early), 8-14 (late), >14 (bedridden)
Postoperative early complication	14

It was observed that 11 patients were mobilized with a permanent prosthesis within one year postoperatively. The mean postoperative time to permanent prosthesis was 104 days. Prolonged postoperative wound discharge developed in 11 patients; five wound discharges regressed with daily dressing. Debridement was performed in six of them due to wound infection. The wounds of four patients healed after debridement, one patient required grafting for wound closure, and amputation revision was performed in one patient.

**Figure 2:**
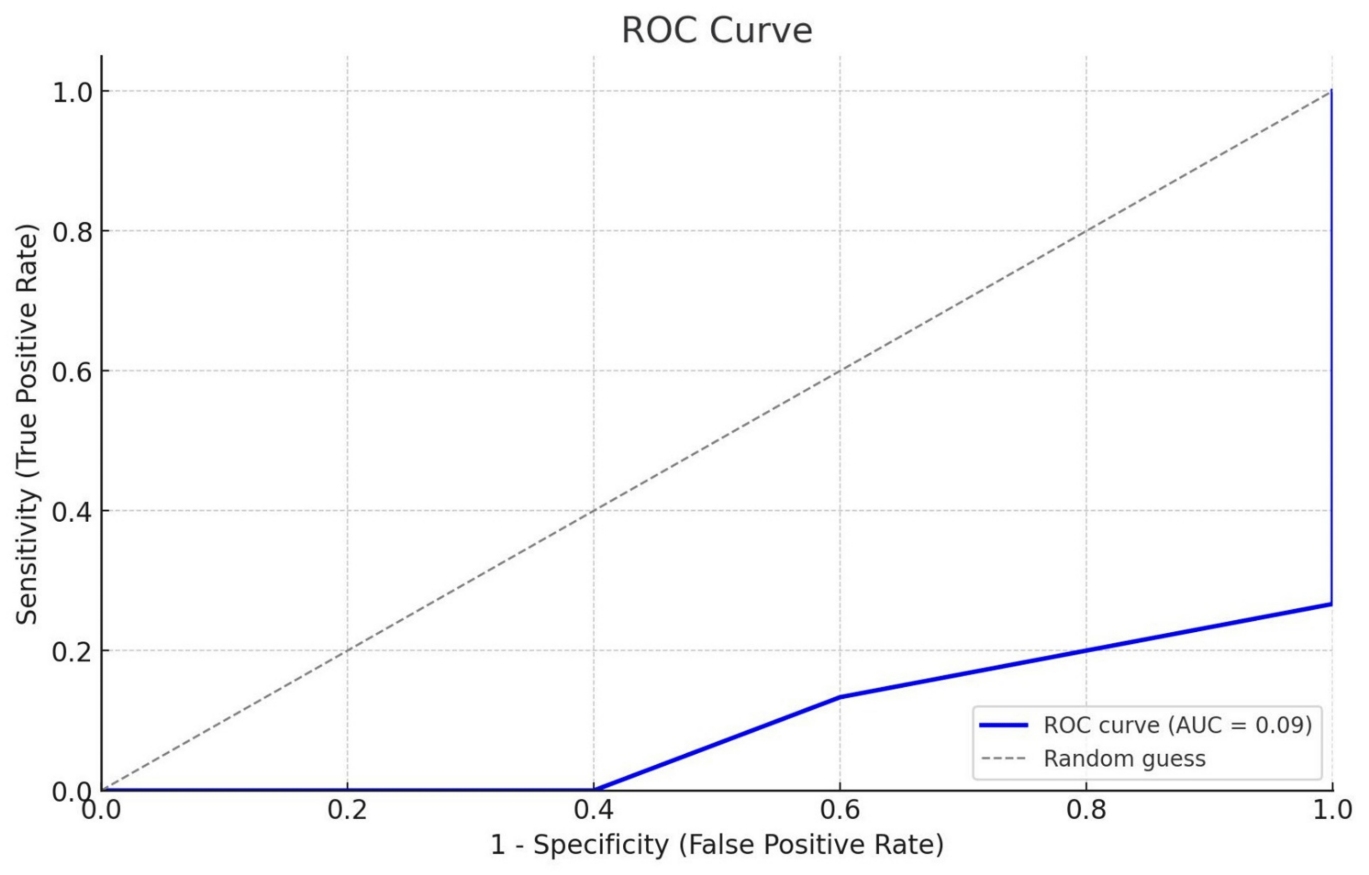
ROC curve for the developed scale predicting one-year mortality ROC: receiver operating characteristic; AUC: area under the curve

According to the scores given to the patients, their survival status, postoperative complications, prosthesis use, and complication status were compared (Table 3). According to the results, the score levels given to the patients differed significantly according to the death status of the patients. As the score level increased, mortality increased (p<0.05). However, ROC analysis revealed low discriminative accuracy for predicting mortality (AUC=0.09), indicating that the scoring system has limited predictive capability regarding mortality outcomes (Figure 2).

**Table 3:** Postoperative one-year follow-up data of patients

Variable	Situation	n	Median	25th-75th percentile	U	p
One-year survival status	Survived	15	5	4.0-6.5	-3.85	<0.001
	Deceased	15	2	1.0-4.0		
Postoperative comorbidity	Absent	20	3	1.0-4.0	-2.63	0.008
	Present	10	5	4.0-7.0		
Prosthetic usage	Absent	19	4	2.0-6.0	-1.33	0.183
	Present	11	4	1.0-5.0		
Postoperative wound complications	Absent	11	4	2.5-5.5	-0.633	0.527
	Present	19	3.5	2.0-4.0		

A strong negative correlation was found between the developed scale and the Katz index of independence in activities of daily living at three months (r=-0.757; p<0.001), indicating that as the score on the developed scale decreased, activities of daily living increased.

In addition, the score levels of the patients differed according to the levels of additional systemic disease (postoperative myocardial infarction, stroke) that developed after the operation (p<0.05). As the scores of the patients increased, postoperative comorbidity also increased. On the other hand, no significant difference was found between the scores of the patients and postoperative prosthesis use and wound complications (p>0.05).

**Figure 3:**
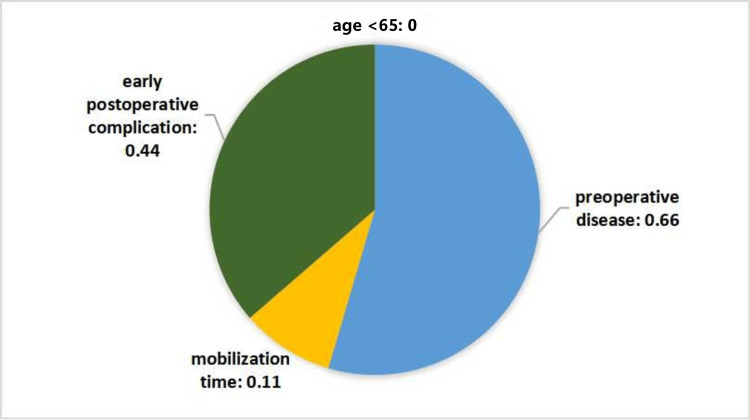
Distribution of factors affecting the mean score of patients aged under 65 years old

Low mortality and high third-month Katz score were seen in below-knee amputation cases 64 years of age and younger. In our scoring, we observed that those aged 64 and younger had a lower mean score [1,19] than other age groups, consistent with the clinic. We concluded that the factors affecting the low scoring are the young age of the patients and the high early mobilization rate (Figure 3). Comorbidities were controlled in our analysis to minimize confounding effects and did not significantly impact the observed trends.

**Figure 4:**
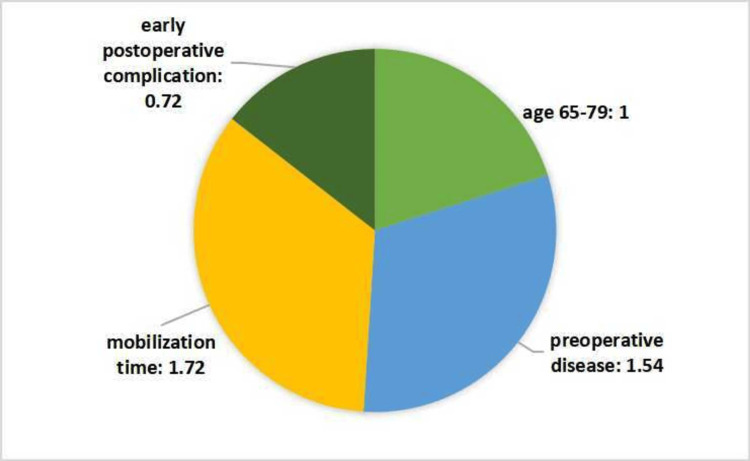
Distribution of factors affecting the mean score of patients aged between 65 and 79 years old

High mortality and low Katz score were seen in patients aged 65-79 years. It was seen that the leading reasons for this situation were low early mobilization, a high rate of preoperative systemic disease, and extremity problems (Figure 4). We saw that they got a high score (mean 5) in the scoring system we used and that our system was compatible with the clinic.

**Figure 5:**
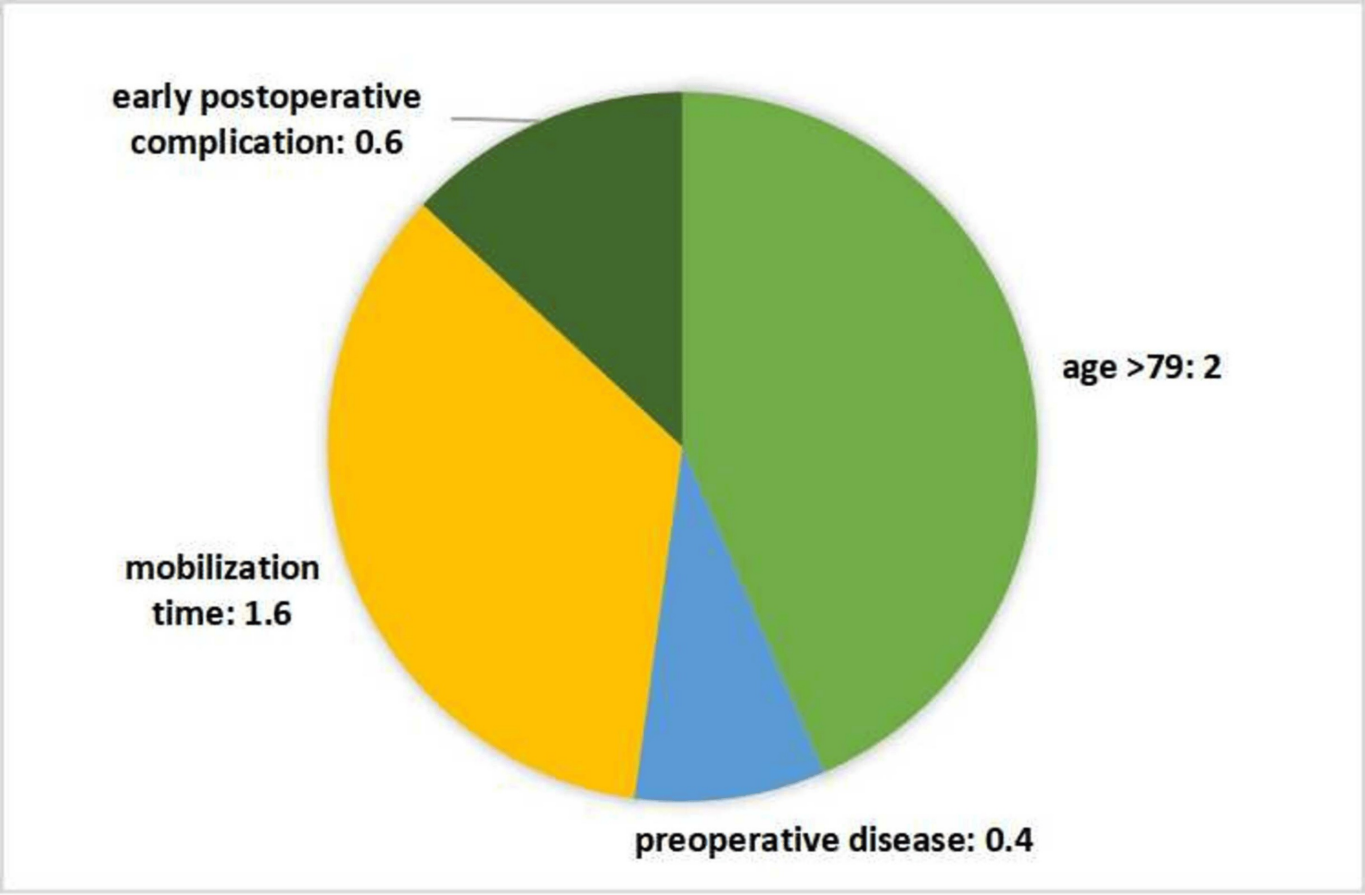
Distribution of factors affecting the mean score of patients aged over 79 years old

High mortality and low Katz score were seen in patients aged 80 and over. When we look at the reason, it was seen that the patient's age was advanced and the rate of immobilization was high (Figure 5). It was seen that these reasons created a high score (mean 4.6) in our scoring and were compatible with the clinic.

## Discussion

The most important finding of this study was that our new amputation scoring system had a good predictive value for mortality and morbidity. The scoring method highlighted the importance of early mobilization based on age and systemic disease, as well as early complications such as hypoxia and metabolic disease.

When we look at the previous literature study on the effect of the factors used on mortality in our scoring system, a study showed that the assessment of perioperative risks in elderly patients undergoing orthopedic surgery should be classified according to comorbidity burden and type of procedures, as well as patient age [13]. In this study, it was stated that while age was an important factor, it was also stated that age groups created differences within themselves. In our study, we classified according to patient age and took age as a factor.

As in many other medical conditions, mortality was higher in the age group of 65-79. In the context of below-knee amputation, this age group also exhibited a lower third-month Katz score. We found that the leading cause of this situation was a higher rate of preoperative systemic disease and extremity problems in the 65-79 age range (Figure 4). The rate of preoperative systemic disease was lower in patients aged 80 and over (Figure 5). It is possible that the higher mortality rate in diabetic patients aged 65-79 with comorbidities may contribute to a lower number of patients surviving beyond the age of 80. However, further studies are needed to confirm this hypothesis. This showed that age is a factor, but it should be evaluated together with other factors as in our scoring. We created our scale by scoring the age groups differently within themselves.

The ROC analysis demonstrated that while the developed scale had limitations in predicting one-year mortality, with an AUC of 0.093, it also highlighted important aspects of patient outcomes that are valuable for clinical practice. The scale's current form shows room for improvement. The modest performance in predicting mortality suggests that some variables, such as the weighing of comorbidities or the time to mobilization, may need further refinement to capture the more complex interplay of factors influencing long-term outcomes. Additionally, incorporating other relevant predictors, such as nutritional status, socioeconomic factors, or psychological resilience, could enhance the scale's predictive capacity. While the AUC result was lower than expected, the scale still offers practical clinical utility, particularly in helping identify patients at higher risk for postoperative complications and those who may require more intensive rehabilitation and follow-up care. With future iterations and the inclusion of more comprehensive variables, the scale holds potential to become a more reliable predictor of long-term outcomes in patients undergoing below-knee amputation.

The strong negative correlation between the developed scale and Katz independence scores at three months indicates that as the score on the scale decreases, the level of independence in daily living activities increases. This relationship is consistent with findings in the literature, where early and effective postoperative care has been shown to significantly improve functional independence in amputee patients. Madsen et al. demonstrated that early mobilization in dysvascular lower-limb amputees is strongly associated with enhanced functional recovery and increased independence. Their systematic review highlighted that patients mobilized within the first 24 hours post-surgery showed greater improvements in daily functional tasks compared to those who experienced delayed mobilization. Similarly, Spaan et al. reported that structured rehabilitation programs incorporating early mobilization are closely linked to improved functional outcomes and greater independence in lower-limb amputees, as reflected by higher Katz scores. These findings emphasize the critical role of early intervention in promoting functional independence in postoperative care for amputee patients [19,20]. In light of this information, we classified mobilization in the first 24 hours as early mobilization and after 24 hours as late in our study. It was observed that the effects of immobilization, late mobilization, and early mobilization on the patient were different both in our clinic and in the literature. In light of this information, we thought and applied that each of them should be scored differently in the scoring system we made. We found that our results were compatible with our application.

In another study noting the postoperative complications of age, elderly patients aged ≥75 years after knee surgery showed a higher incidence of major complications [21]. Similarly, we observed postoperative wound problems, myocardial infarction, and stroke in our one-year follow-up. Eight out of 10 patients with myocardial infarction or stroke died within one year of follow-up. The scoring levels of the patients also differed according to the comorbidity levels developed after the operation (p<0.05). As the scores of the patients increased, postoperative comorbidity also increased.

The American Society of Anesthesiologists (ASA) score is a scoring system that evaluates preoperative anesthesia and is used to predict mortality in patients [7]. Hypertension, chronic obstructive pulmonary disease, and stroke are preoperative systemic diseases that affect the ASA score. In our study, we also included preoperative systemic diseases in our scoring system.

When the effect of diabetic foot infection on mortality was examined, it was stated in the literature that the death rate after diabetic foot infection increases and mortality increases significantly after a large amputation. The findings highlight the importance of early wound and ischemia management for the prevention of diabetic foot infection [8].

We grouped the age factor as 64 years old and under, 65-79 years old, and 80 years old and over. We defined postoperative mobilization as early mobilization, late mobilization, and immobilization (bedridden). We have seen both in the literature and in our clinical results that each of these parameters should be scored differently. In our scoring, we tried to reveal their relationship with mortality more objectively by specifying these subgroups and scoring them differently.

When the data in our study were examined in light of these articles, we saw that important factors for mortality as age progresses are preoperative additional systemic disease and immobilization (bedridden). Another factor in mortality was hypoxia or metabolic disorders, which are early complications. It was observed that early mobilization had a positive effect on the third-month Katz score and reduced mortality.

In a study on mortality with hypoxia in the literature, it was seen that moderate/severe postoperative hypoxemia in the first three days was independently associated with an increase in one-year postoperative mortality [11,22]. The metabolic disorders used in scoring have been shown to increase operative and cardiovascular mortality in patients with multiple metabolic risk factors [16].

A consensus scoring system for patient mortality and morbidity after below-knee amputation has not been found [3]. However, there are various current studies on this subject [4]. We did not use parameters whose effects on mortality and return to social life are controversial in the literature [23,24]. In addition, as the parameters increased, there would be a departure from the simplicity and easy understandability expected from a score. We focused on the parameters that we think are effective according to our clinical experience and literature. After investigating the effects of these parameters in larger patient groups, we think that our scoring can be revised without detracting from being simple and practical.

This study has several limitations. The small sample size of 30 patients may limit the generalizability of the findings to larger populations. Additionally, the retrospective design introduces potential biases, including incomplete documentation and selection bias. The scoring system, while simple and practical, may not account for other relevant factors such as nutritional status, psychological health, or socioeconomic conditions that could influence outcomes. Future studies with larger cohorts and a prospective design are needed to validate and improve the predictive accuracy of the proposed scoring method.

## Conclusions

Our novel scoring system demonstrates a significant correlation with functional independence, highlighting its usefulness for predicting functional outcomes after below-knee amputation. However, due to its low accuracy in predicting mortality, as evidenced by ROC analysis, the scoring method requires substantial refinement. Future studies involving larger patient populations and additional predictive factors are necessary to improve the accuracy and clinical applicability of this scoring approach.
